# Theta-Burst Stimulation of Forearm Muscles in Patients With Complex Regional Pain Syndrome: Influence on Brain and Clinical Outcomes

**DOI:** 10.3389/fpain.2021.736806

**Published:** 2021-09-21

**Authors:** Fannie Allen Demers, Andrea Zangrandi, Cyril Schneider

**Affiliations:** ^1^Noninvasive Stimulation Laboratory (NovaStim), Quebec City, QC, Canada; ^2^Neuroscience Division of Centre de Recherche du CHU of Québec – Université Laval, Quebec City, QC, Canada; ^3^Faculty of Medicine, Université Laval, Quebec City, QC, Canada; ^4^Department of Rehabilitation, Université Laval, Quebec City, QC, Canada

**Keywords:** CRPS, rPMS, TMS, chronic pain, plasticity, neurostimulation

## Abstract

**Objective:** Complex regional pain syndrome (CRPS) is a common pain condition characterized by the changes in the brain that are not yet addressed by conventional treatment regimens. Repetitive peripheral magnetic stimulation (rPMS) of muscles is painless and non-invasive and can influence these changes (the induction of brain plasticity) to reduce pain and improve motricity. In patients with CRPS, this open-label pilot study tested rPMS after-effects on the pain intensity and sensorimotor control of the upper limb along with the excitability changes of the primary motor cortex (M1).

**Methods:** Eight patients with CRPS were enrolled in a single-session program. Patients were tested at pre- and post-rPMS over the flexor digitorum superficialis (FDS) muscle. The clinical outcomes were pain intensity, proprioception, active range of motion (ROM), and grip strength. M1 excitability was tested using the single- and paired-pulse transcranial magnetic stimulation (TMS) of M1.

**Results:** In our small sample study, rPMS reduced instant and week pain, improved proprioception and ROM, and reduced the hemispheric imbalance of several TMS outcomes. The more M1 contralateral to the CRPS side was hyperactivated at baseline, the more pain was reduced.

**Discussion:** This open-label pilot study provided promising findings for the use of rPMS in CRPS with a focus on M1 plastic changes. Future randomized, placebo-controlled clinical trials should confirm the existence of a causal relationship between the TMS outcomes and post-rPMS decrease of pain. This will favor the development of personalized treatments of peripheral non-invasive neurostimulation in CRPS.

## Introduction

Complex regional pain syndrome (CRPS) is formerly known, among others, as reflex sympathetic dystrophy or algoneurodystrophy and is a neuropathic pain characterized by pain arising in one or more limbs, which is disproportionate to an inciting event (fracture, sprain, surgery, or no identification of traumatism), in combination with trophic changes and sensory, motor, and autonomic disorders (1, 2). The causes leading to CRPS remain unclear, but evidence proposes that three mechanisms could explain the symptoms: peripheral changes and sensitization, the dysregulation of the sympathetic nervous system, and maladaptive neuroplasticity (1, 3). The interindividual variability of the contribution of each mechanism over time makes it difficult to administer an efficient treatment for all people with CRPS.

Conventional treatments include medical interventions (medication, topical cream, injections, etc.), rehabilitation (physical and/or occupational therapies), and, ideally, psychological therapy and follow-up. However, despite clinical guidelines and a medical follow-up of the response to treatment, there is almost no evidence to support the therapies currently used in CRPS (4, 5). Approximately 15–20% of people with CRPS have pain and severe related disorders, and 31% are not back to work 2 years after the onset of symptoms (6–9).

The persistence of symptoms and refractoriness to treatment could be due to the central changes that are not sufficiently influenced by conventional approaches, such as changes of volumes, connectivity, activation, and excitability of different parts of the brain (10–19). Among these changes, an imbalance of hemispheric activity between primary motor cortices (M1) has been pointed out (10, 13, 14, 16, 20–23). However, the direction of the imbalance remains controversial: is M1 contralateral to the CRPS hand (M1_contra controlling the limb with CRPS) hyperexcitable or hypoexcitable as compared to M1 ipsilateral (M1_ipsi controlling the non-CRPS hand)?

One new technology currently proposed in clinical research on CRPS is non-invasive repetitive brain magnetic neurostimulation, which is painless and can sustainedly inhibit or excite sensorimotor areas (depending on the used parameters), thus influencing neuroplasticity related to pain and motor improvement (24). However, the excitability imbalance between M1_contra and M1_ipsi can differ between people with hand CRPS (10, 13, 14, 16, 20–23), which questions the rationale of studies that all administrated excitatory neurostimulation over the M1_contra. Another limitation is that people with CRPS experience kinesiophobia (25), i.e., the fear of movement that can generate pain, thus they neglect the use of the CRPS limb, which impairs further motor control due to a reduced generation of proprioceptive information. Thus, a relevant therapy in CRPS could be a bottom-up approach able to increase the flows of proprioceptive signals to the brain to influence neuroplasticity and the mechanisms of pain and motor control. This could be an alternative to the top-down brain stimulation whose rationale in stimulating one hemisphere or another remains unclear.

One such bottom-up approach of interest in CRPS is the repetitive peripheral magnetic stimulation (rPMS), which is applied by means of a coil positioned on the skin over a muscle belly (26). The intensity is suprathreshold to trigger muscle contraction and mimic the mechanisms of muscle contraction/relaxation: this generates flows of proprioceptive information to the brain from the stimulated structures (afferents, muscle fibers, and nerve terminals) but also from the muscles stretched by the joint movement (24, 27, 28). rPMS in physiopathology (the coil over a painful area or over spastic paretic muscles) has been shown to over the changes of frontoparietal networks activity (29) and of M1 excitability (30–33). This was reported with clinical significance, i.e., pain reduction and motor improvement in people with chronic low back pain (30, 31) or with a brain lesion (26, 32, 34, 35), and enhancement of perceptual-cognitive function (36). Furthermore, it was shown in electroencephalography (EEG) studies that somatosensory potentials (SEP) evoked in the primary sensory areas (S1) by suprathreshold rPMS were of pure proprioceptive origin, i.e., with a negligible cutaneous and nociceptive inflow as compared to SEP evoked by peripheral electrical stimulation (37, 38). It is acknowledged that rPMS is painless and influences the sensorimotor areas with a minimal cutaneous contamination of the proprioceptive signals (24). The influence of rPMS at the cortical level could be explained in terms of long-term potentiation (LTP) and long-term depression (LTD), i.e., two mechanisms of neuroplasticity (39, 40), which are already known to be involved in CRPS central sensitization (41). Thus, rPMS that influences M1 excitability could also rebalance hemispheric activity in CRPS and favor central desensitization, all contributing to a decrease in pain.

The present open-label pilot study aimed at testing whether a single rPMS session in people with hand CRPS type 1 (no nerve lesion) could decrease pain (primary outcome) and improve the function of the affected hand. This was tested on rPMS administrated over the flexor digitorum superficialis (FDS) muscle and studied in relation to the excitability changes of hand M1.

## Methods

### Participants and Procedures

Eight people (55.7 ± 9.72 years old, see Table 1) diagnosed with unilateral CRPS type 1 by means of the Budapest clinical criteria (42) were enrolled in an open-label single session of rPMS after signature of the informed consent form approved by the local ethical research board. They were recruited after the discharge from a pain clinic follow-up. The exclusion criteria included spine surgery, major circulatory/respiratory/cardiac diseases, neurological conditions other than CRPS, severe upper limb orthopedic condition, cognitive disorder interfering with the tasks of the study, any history of specific repetitive motor activity (e.g., a musician or professional athlete), and risks related to transcranial magnetic stimulation (TMS) safety guidelines (43, 44). A physician performed medical evaluation at pre-enrollment to confirm eligibility and at post-enrollment to monitor adverse effects. The same physical therapist evaluated all participants to avoid inter-evaluator variability. All data were collected in a single 3-h session (including breaks) on both sides of each participant as follows: first the clinical outcomes and TMS outcomes, then rPMS administration over FDS, 10 min later post-rPMS recollection of TMS data first, and finally the clinical data. All participants were phoned 2, 7, and 30 days later to document any adverse effect (44), and the mean week pain intensity was questioned at day 7 (1-week follow-up). Experimenters analyzing the data remained blind to file codification (pre- vs. post-rPMS) until the completion of analyses.

**Table 1:** General characteristics of participants.

Number (*N*)	8
Age (years): mean ±SD (range)	55.7 ± 9.7 (35–65)
Dominance (*N*: right/left)	7/1
Gender (*N*: females/males)	5/3
Altered side (*N*: right/left)	7/1^*^
Inciting event (*N*: fracture of hand/forearm/arm)	4/3/1
Time since onset of CRPS (months): mean ± SD (range)	41.5 ± 47.8 (12–155)

* *7 right CRPS = 6 right-handers + 1 left-hander, 1 left CRPS = 1 right-hander*.

### Repetitive Peripheral Magnetic Stimulation

Participants were comfortably seated in a reclining-adjustable chair with limb supports and the forearms in supination. rPMS was administered on the CRPS side with an air-film cooled figure-of-eight coil (7 cm outer diameter per wing, a biphasic waveform, 400-μs pulse width, Rapid^**2**^ Magstim, Magstim Company, England) held tangentially on the skin overlying the FDS muscle belly with the long axis of the coil junction perpendicular to muscle fiber orientation [for a review of the parameters of rPMS application, See (24)]. FDS was considered as the target muscle because of a decrease of fingers/wrist flexion active range of motion (ROM) commonly reported in CRPS with a functional significance, such as grasping impairment (6–9). rPMS was delivered at a theta-burst frequency (5-Hz trains of three pulses at 50 Hz during 200 s, 2 s ON/8s OFF, 600 pulses in total). This intermittent mode of rPMS was used to elicit cyclic muscle activation/relaxation as already reported by our research group (32, 34, 45, 46). rPMS intensity was set at 42% of the maximal stimulator output (the maximal intensity on the TBS mode with Rapid^**2**^ Magstim equipment) to produce palpable FDS contractions with visible wrist/finger flexion.

### Clinical Testing

Outcomes were collected only on the CRPS side before and after rPMS of FDS.

*Pain intensity (instant pain and week pain)*: the sitting position of participants was standardized against the backrest of a chair with the shoulder in neutral rotation and 0° abduction, the elbow in 90–100° flexion, and the forearm resting on a height-adjustable therapeutic table. The *visual analog scale* (VAS) was used to quantify the pain intensity experienced by each participant with CRPS. VAS comprises a two-sided band: on one side, the participant uses a cursor to rate the level of pain intensity between the two extremes called “No pain” and “Maximal pain imaginable”, on the other side of VAS, hidden from the participant, a graduation scale from 0 (no pain) to 100 mm (maximal pain imaginable) is used by the evaluator to quantify pain intensity. VAS was used to quantify instantaneous pain at pre- and post-rPMS, the mean pain of the week before rPMS session and the mean pain of the week after the rPMS session (follow-up).

*Active range of finger motion (ROM)*: active ROM in flexion was measured using a manual finger goniometer at the metacarpophalangeal (MCP) and proximal interphalangeal (PIP) joints of the “worst” finger, i.e., the finger (between the third, fourth, and fifth) the harder or more painful to move for each participant. To this end, the elbow was bent (±130°) at rest on a therapeutic table, the shoulder slightly bent (±45°), the wrist and MCP joints in the neutral position (claw hand). Three trials were averaged for per MCP and per PIP. Participants rested for 60 s in between the trials.

*Grip strength*: participants were asked to sit without the support of no arms, with the forearm in the neutral position, and with the wrist between 0 and 30° extension and 0–15° ulnar deviation. The maximal grip strength per hand was measured using a JAMAR hydraulic hand dynamometer (Sammons Preston Rolyan, Bolingbrook, IL, USA). Following the instructions of Mathiowetz (47), the JAMAR dynamometer was set to the second handle position (placed in a hand with the help of the evaluator if needed). The average of three trials per hand was used to represent the maximum grip strength of each participant. Standardized cheers were given (“squeeze hard, hard, hard, the hardest, and release”) over 8 s of the sustained contraction to promote a reliable value (48). Participants took rest for 30 s in between the trials. To ensure the validity of interindividual comparisons, grip strength was expressed in percentages of norms stratified by age, sex, and side (right/left hand) (47).

*Upper limb proprioception*: following the guidelines of Le Métayer, the paradigm of the direct and blurred proprioception of the upper limb was used to assess the ability to perceive the limb position in space (49). Briefly, the participants were seated, leaning against a chair without armrests and bending the knees at 90° with feet flat on the floor and hands on the thighs (initial position). They had first to reach with the index a target (a large red dot) on a graduated screen at gaze height and arm distance in front of them and return to the initial position and to repeat this practice three times. Then, they were instructed to close the eyes. For the direct proprioception, they had to reach back the target with the index (one trial only) with the eyes closed. For the blurred proprioception, the evaluator cautiously (without pain) moved the tested arm in the air with shoulder movements and elbow flexion/extension (to perturb the position proprioceptive reference) and put the arm back in the initial position, and the participants, without opening the eyes, had to reach back the target with the index (one trial only). The respective scores of the direct and blurred proprioception were the distance (measured in centimeters) between the finger contact on the graduated screen (with the eyes closed) and the target.

### Transcranial Magnetic Stimulation Testing

Transcranial magnetic stimulation procedures were strictly replicated for each hemisphere before and after the rPMS of FDS, following the guidelines from the International Federation of Clinical Neurophysiology (50). TMS was applied over the first dorsal interosseous (FDI) muscle M1 area whose excitability can be modulated by the FDS M1 area recruitment owing to a proximo-distal synergy in M1 (51, 52).

*Surface electromyographic (EMG) recordings*: participants were comfortably seated in a reclining chair with arms and legs supported and hips and knees slightly bent (20° flexion). After the standard skin preparation (i.e., cleaning the skin with alcohol) (53), the parallel-bar EMG sensors with adhesive skin interfaces were installed on each participant (a fixed distance of 1 cm between the electrodes, 16-channel Bagnoli EMG System, Delsys, Inc., Boston, MA, USA). EMG sensors were placed bilaterally on the FDI belly. A common ground electrode was placed on the ulna olecranon of the tested limb. EMG signals were band-pass filtered (20–450 Hz), amplified before digitization (2 kHz), and computer-stored for an offline analysis (PowerLab acquisition system, LabChart, ADInstruments, Colorado Springs, CO, USA). EMG procedures were followed without inducing pain in participants with CRPS.

*Hotspot*: magnetic stimuli were applied over the FDI M1 area by a figure-of-eight coil (7-cm outer diameter each wing, Magstim Company Limited, Whitland, UK) positioned on the scalp with the long axis of the two-wing intersection pointing antero-posteriorly at a 45° angle from the medial line to active M1 cells in the postero-anterior direction (54). The 10–20 EEG system was used to first approximate the FDI M1 area (55). The position was then slightly adjusted to determine the “hotspot,” namely, the M1 location where the motor evoked potential (MEP) obtained in the contralateral FDI was of the highest amplitude at the lowest TMS intensity (56). This hotspot was marked on the scalp (surgical pen) to ensure reliable coil positioning across the time of testing (56), and real-time EMG recordings from both sides had helped to monitor the complete relaxation of FDI during TMS testing.

*Resting motor threshold (RMT)*: RMT was determined at the TMS intensity that elicited at least 5 FDI MEPs with an amplitude of 50 μV or higher out of 10 trials. RMT expressed in percentage of the maximal stimulator output (% MSO) has good validity and reliability (33, 57, 58) and provided information on the basic excitability of M1 (50, 59). The difference of RMT between hemispheres was calculated, and its absolute value represented the hemispheric balance.

*MEP amplitude and latency*: a suprathreshold test (unconditioned) TMS at an intensity of 120% RMT enabled to collect and measure the test MEP amplitude and latency. Test MEP amplitude (in mV) measured peak-to-peak informs on the volume of M1 cells activated by TMS and the excitability of the corticospinal pathway (50, 60, 61). Test MEP latency (ms) measured from TMS artifact to MEP onset it reflects conduction time and indirect M1 cell synchronization by TMS and the synchronicity of descending volleys to depolarize spinal alpha-motoneurons (61, 62).

*M1 inhibition and facilitation*: paired-pulse TMS (coil connected to two Magstim 200^2^ monophasic stimulators) was used to test the activity of M1 circuits of short-interval intracortical inhibition (SICI), intracortical facilitation (ICF), and short-interval intracortical facilitation (SICF), which provided information on different aspects of M1 function (63–65). In SICI, a subthreshold conditioning TMS (80% RMT eliciting no MEP on its own) was delivered 3 ms before the test TMS at 120% RMT (65). In ICF, the same parameters were used but at an inter-stimulus interval of 15 ms (65). The conditioned MEP expressed *post hoc* relative to the mean test MEP amplitude is usually of lower amplitude than the test in SICI and of higher amplitude in ICF (65). SICI probes the function of GABA_A_ interneurons within M1, and ICF probes the function of oligosynaptic glutamatergic circuits (50, 59). In SICF, two stimuli were delivered 1 ms apart, the first TMS at 100% RMT and the second at 90% RMT. MEP amplitude was then expressed relative to the amplitude of test MEP at 100% RMT, which likely reflects I-wave summation following the depolarization of M1 interneurons by TMS and probing the function of short circuits of glutamategic interneurons around the corticospinal cell somas (50, 59, 63). The test TMS intensity was adjusted at post-rPMS to match the amplitudes of the test MEP obtained at pre-rPMS for the validity of comparisons of conditioned MEP amplitudes between pre- and post-rPMS (32). About 12 test MEP and 12 conditioned MEP were collected per participant at a frequency of 0.2–0.3 Hz with 10% variations between the trials. Breaks were encouraged and given on request.

### Data Reduction and Statistical Analysis

Seven clinical outcomes were tested pre- and post-rPMS in all participants for the CRPS side: instant and week pain intensity (mm), active MCP and PIP joint ROM of the worst finger (degrees), grip strength (percentage norm), and direct and blurred proprioception (cm). Bilateral paired Student's *t*-tests applied to each variable assessed the clinical changes obtained between pre- and post-rPMS.

Six TMS outcomes were collected for each hemisphere at pre- and post-rPMS: RMT (% MSO), MEP amplitude (mV), MEP latency (ms), SICI, ICF, and SICF (percentage test). The changes were tested by means of repeated measure ANOVAs (ANOVA_RM_) with factor time (pre- vs. post-rPMS) and hemisphere (M1_contra vs. M1_ipsi) applied on each outcome. *Post*-*hoc* tests were not corrected for multiple comparisons, the correction being unnecessary for an explorative analysis (66–68). Bilateral paired Student's *t*-tests were applied to the hemispheric imbalance of each TMS outcome (the absolute value of the between-hemisphere difference) to detect any rebalance after rPMS.

Shapiro–Wilk's test for normality revealed that most data were normally distributed, thus parametric tests were used. The maximum norm residual test (Grubbs' test) had helped to detect an outlier from a data set approximated by normal distribution (Prism 8.0, GraphPad Software, Inc., La Jolla, CA, USA): data set is analyzed and an outlier would typically be expunged when it falls beyond a critical deviation from the sample mean (chance probability to obtain the outlying value < 0.05). The significance level was set at *p* < 0.05. Statistical analysis was conducted using SPSS Stastistics (version 25). Pearson's correlation matrices were produced to examine the relationships between the change of the primary outcome (pain intensity) and the amount of change or baseline values of other variables.

## Results

### Clinical Outcomes

**Figure 1:**
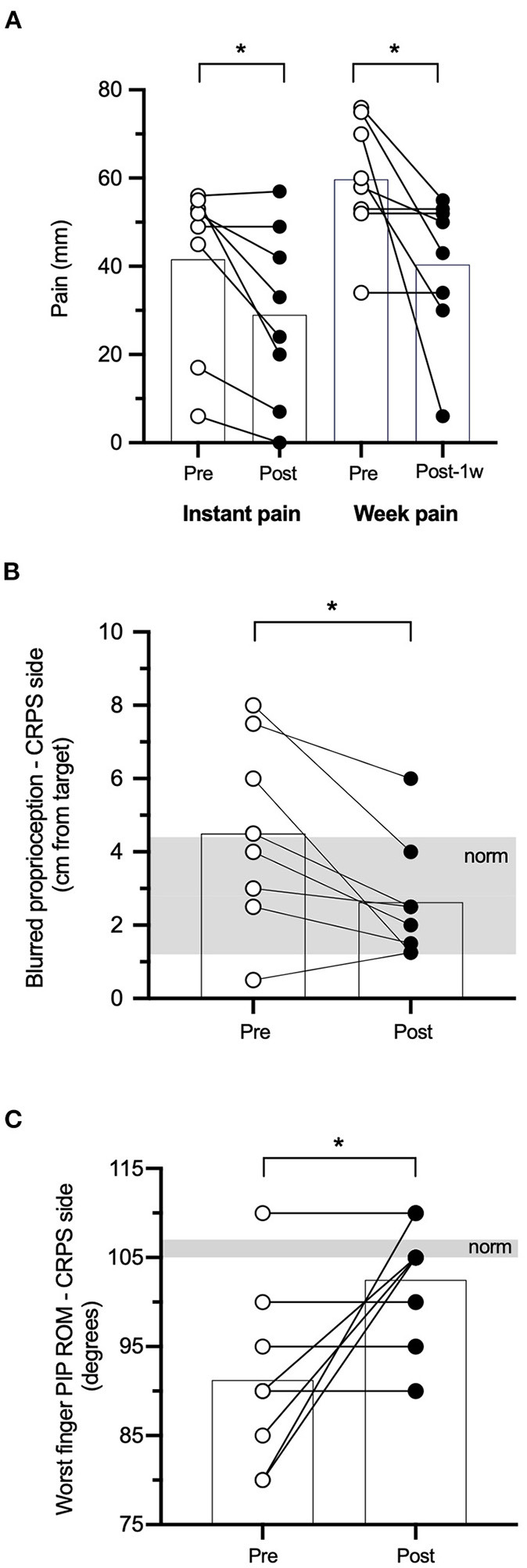
Clinical outcomes. Individual values and group means (superimposed histograms) at pre- and post-repetitive peripheral magnetic stimulation (rPMS) (white and black circles, respectively) of instant and week pain in millimeters of a visual analog scale (VAS) **(A)**, blurred proprioception performance in centimeters from the target **(B)**, and proximal interphalangeal range of motion (PIP ROM, degrees) of the worst finger **(C)**. The shaded area in **(B,C)** represents the clinical norm (mean ± 1 SD). *p < 0.05 between pre- and post-rPMS.

The seven clinical outcomes at pre- and post-rPMS are reported in Table 2 (upper part), and Figure 1 shows the significant changes detected after rPMS.

**Table 2:** Clinical and TMS data of participants.

**Outcome**	**Time**	
	**pre-rPMS (mean ± SD)**	**post-rPMS (mean ± SD)**
**Clinical outcomes** ***—*** **CRPS side only**		
VAS instant pain (mm)	41.63 ± 19.14	**29.00** **±** 20.00^*^
VAS week pain (mm)	59.75 ± 14.01	**40.38** **±** 16.64^*^
D-proprioception (cm from target)	1.63 ± 0.83	1.34 ± 0.64
B-proprioception (cm from target)	4.50 ± 2.56	**2.63** **±** 1.64^*^
Worst MCP ROM (degrees)	76.25 ± 15.06	79.38 ± 18.41
Worst PIP ROM (degrees)	91.25 ± 10.26	**102.50** **±** 7.07^*^
Grip strength (% norm)	72.75 ± 16.22	72.45 ± 18.53
**TMS outcomes—both hemispheres**		
RMT (% MSO)	M1_contra: 55.50 ± 8.78 M1_ipsi: 75.38 ± 7.23 Δ (abs): 7.63 ± 4.34	M1_contra: 54.63 ± 6.59 M1_ipsi: 55.63 ± 5.95 Δ (abs): **±** **4.50** **±** 3.93^*^
MEP amplitude (mV)	M1_contra: 0.60 ± 0.40 M1_ipsi: 0.83 ± 0.90 Δ (abs): 0.47 ± 0.76	M1_contra: 0.63 ± 0.38 M1_ipsi: 0.64 ± 0.55 Δ (abs): 0.35 ± 0.31
MEP latency (% height)	M1_contra: 24.26 ± 0.42 M1_ipsi: 24.55 ± 0.42 Δ (abs): 0.48 ± 0.40	M1_contra: **26.30** **±** 0.88^*^ M1_ipsi: 25.64 ± 2.08 Δ (abs): **1.24** **±** 0.58^*^
Conditioned MEP—SICI (% test)	M1_contra: 99.78 ± 67.02 M1_ipsi: **34.90** **±** **15.03**^**+**^ Δ (abs): 64.88 ± 53.99	M1_contra: 39.19 ± 22.01 M1_ipsi: 98.18 ± 97.06 Δ (abs): 69.64 ± 74.33
Conditioned MEP—ICF (% test)	M1_contra: 168.25 ± 129.63 M1_ipsi: 168.94 ± 108.10 Δ (abs): 147.27 ± 117.30	M1_contra: 177.28 ± 82.94 M1_ipsi: 227.14 ± 113.15 Δ (abs): **54.41** **±** 33.47^*^
Conditioned MEP—SICF (% test)	M1_contra: 605.75 ± 510.96 M1_ipsi: 463.00 ± 554.04 Δ (abs): 538.89 ± 463.39	M1_contra: 455.89 ± 272.88 M1_ipsi: 426.45 ± 272.65 Δ (abs): **228.48** **±** 237.45^*^

*Δ (abs), absolute difference between hemispheres, SD, standard deviation, VAS, visual analog scale, ROM, range of motion, MCP, metacarpophalangeal joint, PIP, proximal interphalangeal, D-proprioception, direct proprioception, B-proprioception, blurred proprioception, RMT, resting motor threshold, MSO, maximal stimulator output, MEP, motor evoked potentials, TMS, transcranial magnetic stimulation, SICI, short-interval intracortical inhibition, ICF, intracortical facilitation, SICF, short-interval intracortical facilitation, M1_contra or M1_ipsi: M1 contralateral or ipsilateral to the CRPS side. Bold values represent significant differences*.

* *p < 0.05 between pre- and post-rPMS*,

+ *p < 0.05 between sides*.

*Pain*: the mean instant pain of the group after rPMS (29.0 ± 20.0 mm) showed a reduction as compared to pre-rPMS (41.6 ± 19.14 mm, *t*_7_ = 2.947, *p* = 0.022; Figure 1A). The mean week pain after rPMS (40.38 ± 16.64 mm) also showed a reduction as compared to pre-rPMS (59,75 ± 14.01 mm, *t*_7_ = 2.443, *p* = 0.045; Figure 1A). The individual data presented in Figure 1A denote that six participants (75% of the group) reported that instant pain after rPMS was reduced (range: 6–35 mm decrease) and five (62%) reported that pain reduction persisted for a week after the rPMS session (range: 8–64 mm decrease).

*Proprioception*: the mean performance of the upper limb in CRPS for the blurred proprioception after rPMS (2.63 ± 1.64 cm, within the norm illustrated by the gray area) showed an improvement as compared to pre-rPMS (4.50 ± 2.56 cm, further from the target than the norm + 1SD, *t*_7_ = 2.958, *p* = 0.021; Figure 1B), i.e., a mean change of pointing almost 2 cm closer to the target. The individual data show that all participants pointed closer to the target after rPMS (range: 1–4.75 cm closer) but one whose baseline performance was closer to the target than the norm itself [2.8 ± 1.6 cm (49)]. Of note, after rPMS, only one participant still presented a performance further from the target than the norm + 1 SD, against four at baseline. The direct proprioception performance remained unchanged after rPMS (see Table 2) and also within the norm [1.9 ± 1.3 cm (49)].

*Range of motion*: the mean worst PIP ROM after rPMS (102.50° ± 7.07°) showed an increase as compared to pre-rPMS (91.25° ± 10.26°, *t*_7_ = −2.496, *p* = 0.041; Figure 1C). The individual data show that the four participants (50% of the group) with baseline values more than 16° and less than the norm increased their ROM after rPMS (range: 15–30°) and even reached the norm or values beyond the norm [norm = 106° ± 1°, (69)]. Of the four other participants who did not show any improvement, two had close to normal (or better) ROM. No change was detected for the worst MCP ROM after rPMS.

*Grip strength*: the grip strength expressed in percentage of the norm relative to age, sex, and side (right/left) remained unchanged between pre- and post-rPMS (see Table 2).

### TMS Outcomes

**Figure 2:**
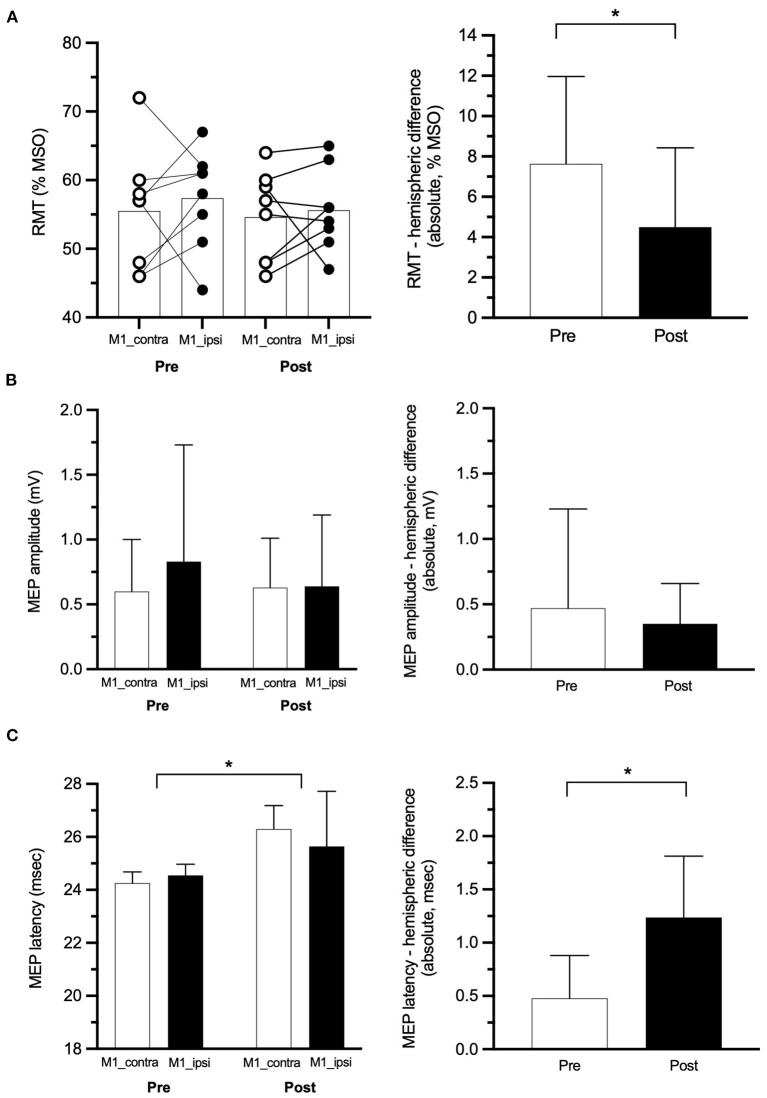
Single-pulse transcranial magnetic stimulation (TMS) outcomes. **(A)** Left panel: individual values of RMT at pre- and post-rPMS with superimposed group means (histograms) for M1 contralateral to the complex regional pain syndrome (CRPS) side (M1_contra: white circles) and M1 ipsilateral (M1_ipsi: black circles), right panel: the absolute difference of RMT between M1_contra and M1_ipsi at pre- and post-rPMS (empty and filled histograms, respectively). Amplitude **(B)** and latency **(C)** of motor-evoked potentials (MEP) at pre- and post-rPMS for M1_contra and M1_ipsi (white and black histograms, respectively) on the left panel and, on the right panel, their absolute between-M1 difference at pre- and post-rPMS (white and black histograms, respectively). RMT, resting motor threshold, MSO, maximal stimulator output. *p < 0.05 between pre- and post-rPMS.

**Figure 3:**
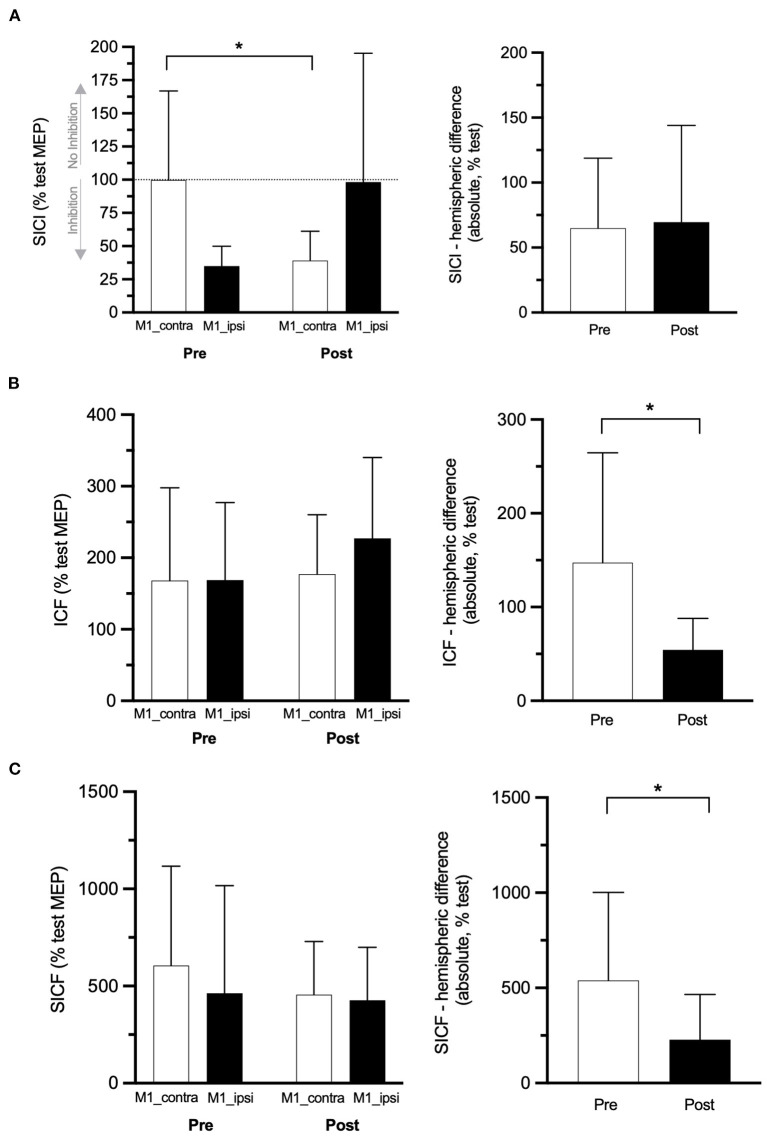
Paired-pulse TMS outcomes. SICI **(A)**, ICF **(B)**, and SICF **(C)** data at pre- and post-rPMS in M1 contralateral to the CRPS side (M1_contra: white histograms) and M1 ipsilateral (M1_ipsi: black histograms) on the left panel and, on the right panel, their absolute between-M1 difference at pre- and post-rPMS (white and black histograms, respectively). SICI, ICF, SICF: conditioned MEP of short-interval intracortical inhibition, intracortical facilitation, and short-interval intracortical facilitation. *p < 0.05 between pre- and post-rPMS (for M1_contra only in **A**).

One participant (female, 58 years old, diagnosed with CRPS 11 months ago, baseline pain of 52 mm on the VAS) reported head discomfort during the application of the paired-pulse TMS paradigms (both hemispheres), and this data collection was ceased. Thus, paired-pulse TMS outcomes (SICI, ICF, and SICF) were collected in *n* = 7 participants at pre- and post-rPMS. This participant was, however, not removed from the analyses of the single-pulse TMS outcomes (*n* = 8 participants for RMT, MEP amplitude, and latency in both hemispheres). No other adverse effect was reported during experiments or follow-ups. The six TMS outcomes at pre- and post-rPMS are reported in Table 2 (lower part) and are shown in Figure 2 (single-pulse TMS) and Figure 3 (paired-pulse TMS).

#### Single-Pulse TMS Data

No effect was detected with the ANOVA_RM_ applied on RMT. Individual data (Figure 2A, left panel) show that, at pre-rPMS, the basic excitability of M1_contra was higher (lower RMT) than that of M1_ipsi in six participants (75%), and the reverse in the other two. For the whole group, the absolute value of this hemispheric difference of RMT after rPMS (4.5 ± 3.9% MSO) showed a decrease as compared to pre-rPMS (7.6% ± 4.3% MSO, *t*_7_ = 2.667, *p* = 0.032; Figure 2A, right panel). An RMT imbalance greater than 4.7–5.3% MSO is considered to be significantly larger than the minimal detectable change (33), and this was observed for five participants at pre-rPMS and for only two after rPMS.

No effect was detected with the ANOVA_RM_ applied on the MEP amplitude (Figure 2B). For MEP latency, ANOVA_RM_ revealed a main effect of time [*F*_(1, 7)_ = 10.353, *p* = 0.015; Figure 2C, left panel] with a longer MEP latency after rPMS than before (see Table 2 for means per hemisphere). The absolute hemispheric difference of MEP latency after rPMS (1.24 ± 0.58 ms) was higher compared to before rPMS (0.48 ± 0.40 ms, *t*_7_ = −2.609, *p* = 0.035; Figure 2C, right panel).

#### Paired-Pulse TMS Data

ANOVA_RM_ applied on the SICI-conditioned MEP amplitude showed a Hemisphere × Time interaction [*F*_(1, 6)_ = 6.284, *p* = 0.046; Figure 3A, left panel]. Of note, a higher amplitude of SICI-conditioned MEP represents a decrease of SICI and *vice versa*, and a value closer to 100% means no SICI. *Post hoc* analyses showed that, in M1_contra, SICI after rPMS (39.19% ± 22.01% test MEP) was increased as compared to pre-rPMS (99.78% ± 67.02%, *t*_6_ = 2.529, *p* = 0.045). The reverse was observed in M1_ipsi, although not reaching significance, with the decrease of SICI after rPMS (98.18% ± 97.06%) as compared to pre-rPMS (34.90% ± 15.03%, *t*_6_ = −1.927, *p* = 0.010). In addition, the amplitudes of SICI-conditioned MEP were different between M1_contra and M1_ipsi at pre-rPMS (*t*_6_ = 0.068, *p* = 0.019) but not at post-rPMS (likely due to a large SD in M1_ipsi). The absolute SICI hemispheric difference (Figure 3A, right panel) was similar between pre- and post-rPMS (*t*_6_ = −0.251, *p* = 0.810), but its direction after rPMS (M1_contra with 59% more SICI than M1_ipsi) was reversed as compared to pre-rPMS (M1_contra with 64.88% less SICI than M1_ipsi).

No effect was detected with the ANOVA_RM_ applied on the ICF- and SICF-conditioned MEP amplitudes (Table 2 and Figures 3B,C, left panels). The absolute ICF hemispheric difference after rPMS (54.4 ± 32.5%) was decreased as compared to pre-rPMS (147.3 ± 111.5%, *t*_6_ = 2.42, *p* = 0.052; Figure 3B, right panel). The absolute SICF hemispheric difference after rPMS (228.5 ± 237.5%) was decreased as compared to pre-rPMS (538.9% ± 90.11%, *t*_6_ = 3.28, *p* = 0.017; Figure 3C, right panel).

#### Correlation: Amount of Pain Change and RMT Hemispheric Difference at Baseline

**Figure 4:**
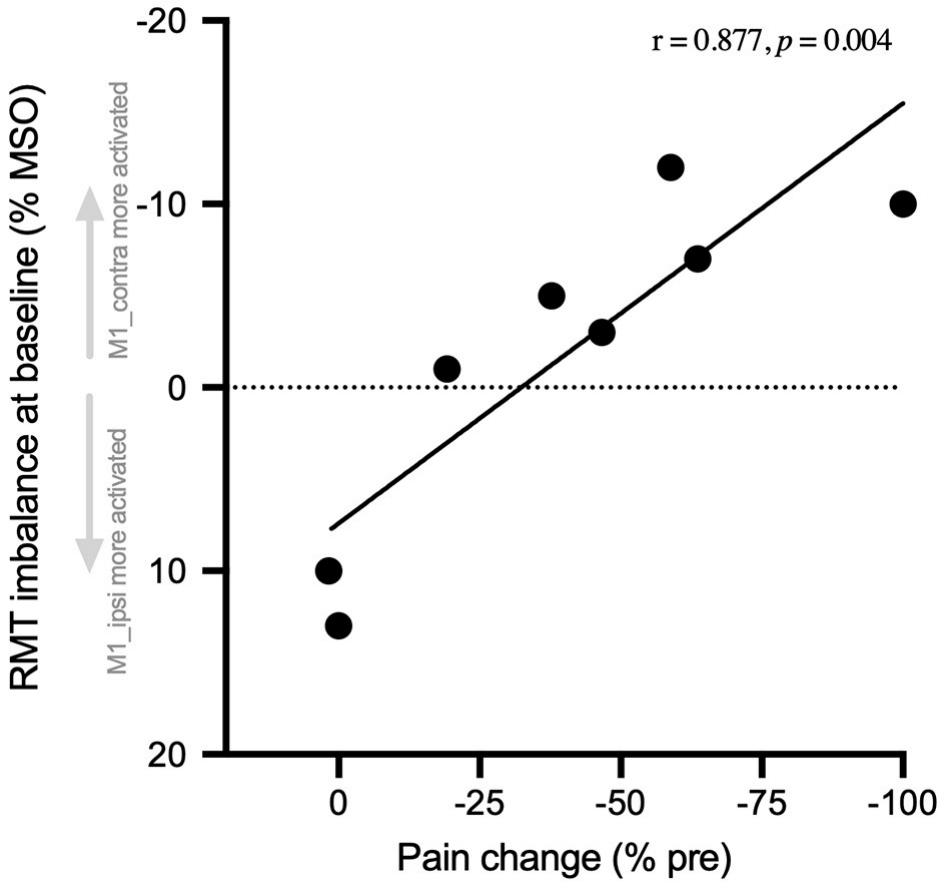
Pain change correlated with baseline RMT hemispheric balance. The number of changes of instant pain at post-rPMS (% pre-rPMS) is expressed against the raw hemispheric imbalance of RMT at pre-rPMS (% MSO). This imbalance was calculated as the difference of RMT between M1 contralateral to the CRPS side (M1_contra) and M1 ipsilateral (M1_ipsi). Thus, the negative values (above 0, Y-axis reversed) indicate the participants with baseline RMT that was lower for M1_contra (i.e., M1_contra excitability higher than M1-ipsi). The more M1-contra was activated as compared to M1_ipsi at baseline, the more instant pain was reduced after rPMS. RMT, resting motor threshold, MSO, maximal stimulator output.

The amount of pre- to post-rPMS change of instant pain (% baseline) was correlated with the raw difference of RMT at baseline between M1_contra and M1_ipsi (*r* = 0.877, *p* = 0.004; Figure 4). Precisely, the greater the negative value of RMT difference (see Y-axis reversed), i.e., the higher the basic excitability of M1_contra as compared to M1_ipsi, the more the instant pain after rPMS decreased.

## Discussion

This open-label pilot study using a pre–post design in eight people with long-term CRPS showed that a single rPMS session over the forearm muscles decreased instant and week pain and improved the hand sensorimotor function, as denoted by the changes of blurred proprioception and PIP joint ROM. These clinical changes were paralleled by mechanisms of M1 plasticity as delineated by the changes of TMS outcomes, namely M1 inhibition, as tested by SICI, and hemispheric balance of RMT, ICF, and SICF. The discussion that follows expands on the potential causal relationship between the TMS outcomes (and their changes after rPMS) and the improvement of the condition of the patients after the rPMS session.

### Clinical Changes After rPMS and Relation to Hemispheric Balancing

#### Pain and Function

Only one study, before ours, used rPMS in CRPS but collected only TMS outcomes, thus providing no support of clinical significance (70). This study used a single session as ours, but rPMS was applied over the cervical spine (C7-C8) at 20 Hz for a total of 2,000 pulses over 10 min, whereas we used iTBS (50 Hz) over the FDS on the painful side for a total of 600 pulses over 3 min 20 s. Thus, the protocols are not comparable. Our research group has already administered rPMS in the iTBS mode and successively decreased pain and improved posturomotor control in people with other pain conditions such as chronic low back pain (30, 31). The present findings of immediate pain decrease after one session of rPMS, and its persistence over a week support the therapeutic potential of rPMS in the CRPS condition. Furthermore, it is questioned whether repeated sessions of rPMS could have ensued a larger improvement for all outcomes and in all participants. Indeed, as developed in the two next paragraphs, pain was not reduced in some individuals and rPMS did not improve all outcomes.

Individual scores showed that instant pain in two participants was not reduced after rPMS. This could reflect that rPMS presented with an inter-individual variability of after-effects owing to individual factors, the etiology of CRPS, or pain characterization and intensity, etc., which could not be studied as co-variables in this study due to a small sample size. Also, pain was not reduced in three participants after a week post-rPMS. The reason could be still the inter-individual variability. More interestingly, and even if participants were instructed not to change their life habits during the week post-rPMS, it is possible that some used their less painful CRPS side more than before the rPMS session, whereas others did not. The level and duration of practice with the CRPS side over a week may have obviously influenced the duration of rPMS after-effects (further decreasing, for example, pain and kinesiophobia). Future studies may thus have to standardize the physical activities of participants during the follow-ups.

Pain decrease in parallel with an increase in the worst finger PIP active ROM resembles the changes reported after 15–30 sessions of transcutaneous electrical neurostimulation (TENS) (71–74) or after 5 sessions of TENS in combination with brain stimulation (75). Thus, the similarity with our results after only one session of rPMS suggest that rPMS in CRPS was likely more efficient than TENS in CRPS. This could be due to the different nature of sensory information generated by rPMS and TENS. Indeed, EEG studies deciphered that the suprathreshold magnetic stimulation of a nerve elicited magnetic SEP in S1 that were of pure proprioceptive origin as compared to electrical SEP (24, 37, 38), i.e., with negligible cutaneous and nociceptive inflows. In other words, rPMS may have favored the proprioceptive-to-motor transduction required in pain modulation and motor control (24, 37, 38). Future studies will have to test whether repeated sessions of rPMS enlarge the gains obtained after a single session. They will have to test also whether the worst MCP ROM and the grip strength can be improved (unchanged in our study). Indeed, it could be assumed that the performances during the worst MCP ROM and the grip strength were suboptimal due to the fear and fatigue of hand movement (more than for the distal PIP joint ROM): participants may need greater regimens of rPMS (and not only one session) to dissociate movement from pain and entrain functional improvement.

#### Proprioception

Interestingly, our original results on the improved performance in the blurred proprioception paradigm (49) support that rPMS enhanced the perception of the CRPS upper limb in space and its motor control after a postural perturbation. The fact that the direct proprioception was not improved may be related to ceiling effects (performances were within the normative values at pre-rPMS). This also suggests that body perception in CRPS might be impaired only after a perturbation of the reference (blurred proprioception). This is in line with studies on the post-rPMS activation of frontal-parietal pathways involved in movement perception and sensorimotor planning (29) and the enhancement of perceptual-cognitive function of coordinated movement (36). Also, as already suggested in chronic low back pain (31), the increased flows of proprioceptive inputs after rPMS (which are decreased in CRPS due to limb non-use) may have favored the selection of more efficient strategies of pain management, in addition to an action on sensorimotricity and pain control, thus explaining the persistence of pain decrease over a week. In the same vein, future CRPS studies should explore if rPMS can attenuate or extinct the fear of movement. That could be a psychological effect when participants observe their hand movement without pain during rPMS-generated muscle contraction. However, this could be also related to the activation of thalamo-amygdala circuits, which were recently revealed as attenuators of remote fear memories in mice (76). Indeed, rPMS activates the lemniscal pathway (as evidenced by proprioceptive magnetic SEP), thus the thalamus is recruited and such circuits of fear attenuation could be involved in the long-term changes observed in the present study.

#### Hemispheric Balance

Most interestingly, the efficiency of rPMS to decrease pain among participants seemed to depend on the hemispheric balance of M1 excitability measured at baseline using RMT. Indeed, the more M1_contra (contralateral to CRPS side, in CRPS hemisphere) was activated as compared to M1_ipsi prior to rPMS, the greater was the decrease of pain after rPMS (Figure 4). This original finding does not resolve the controversy regarding the direction of M1 imbalanced activity in CRPS, i.e., which hemisphere is more activated than the other (10, 13, 14, 16, 20–23). But, it rather deciphers that, for the first time, beyond inter-individual differences, the direction of M1 activity imbalance matters for the responsiveness to rPMS. One hypothesis could be that the entrainment by rPMS of the proprioceptive-to-motor mechanisms of pain modulation (24, 37, 38) could be more efficient when M1 contralateral to rPMS is basically more activated than its counterpart.

This important result was paralleled by the reduction, after rPMS, of the hemispheric imbalance of RMT itself, but also of ICF and SICF (right part of Figures 2A, 3B,C, respectively). RMT probes the homeostasis of glutamatergic excitation and GABAergic inhibition of M1 cells, and ICF and SICF provide information on the activity of different glutamategic circuits surrounding M1 cells (50, 59, 63). Thus, the reduction of hemispheric imbalance could be related to the remote action of rPMS on the glutamate and GABA receptors at the cortical level, i.e., influencing LTP and LTD mechanisms, as already suggested in chronic low back pain (31) and chronic stroke (34). Given LTP and LTD are involved in CRPS central sensitization (41), rPMS may have favored central desensitization and pain decrease. ICF and SICF hemispheric imbalance has never been studied, and our data cannot be compared to literature. It is known that an RMT difference of more than 4.7–5.3% MSO between hemispheres is a cut-off for a significant imbalance (33). Its reduction to less than these values after rPMS, in parallel with an improvement of the function and of proprioception, could further support the bottom-up action of rPMS to influence the CRPS condition. Of note, the lengthening of the corticospinal latency (Figure 2C) could reflect that the synchronicity of the descending volleys upon the spinal alpha-motoneurons was impaired by some inhibition mechanisms, such as those involved in the reactivation of descending anti-nociceptive pathways.

Larger sampled studies in the future will have to verify whether these results survive, and in particular whether the hemispheric balance of M1 excitability as measured by RMT at baseline can predict the responsiveness to rPMS. The identification of better and lesser responders to rPMS will be of clinical importance to test rPMS therapeutic efficacy in CRPS.

### Influence of rPMS on M1 Inhibition in CRPS

rPMS can influence M1 excitability because the massive flows of somatosensory signals mediated to the brain can drive M1 plasticity (77). For example, neuroimaging techniques and TMS studies reported that in motor disorders rPMS influenced the contralateral M1 excitability and the frontoparietal networks activity *via* the activation of lemniscal, thalamo-cortical, and spino-cerebellar pathways, thus enhancing the motor function (24, 29, 78, 78). However, this had never been studied in CRPS concurrently to clinical changes.

In our study, rPMS on the CRPS side significantly increased SICI, which was missing at pre-rPMS in M1_contra (Figure 3A, left part). The lack of SICI in M1-contra at baseline is in line with the literature reporting M1 disinhibition in the CRPS hemisphere (16, 79). However, our original findings support for the first time that the clinical changes on the CRPS side could have been driven by the reactivation of SICI circuits in the CRPS hemisphere. SICI is characterized as a fast dynamic inhibition mediated by inhibitory interneurons surrounding the soma and dendrites of corticospinal cells and acting on GABA_A_ receptors (80). SICI helps to tune the movement-related neuronal activity to shape sensorimotor programs, including the planning of pain modulation, and segregates M1 circuits for the selection of strategies or sequences of action appropriate for the target task, such as, for example, syncopated or synchronized finger movements or interjoint synergies (52, 81). This explains, at least in part, the presence of pain and motor issues when SICI is missing, such as in CRPS (16, 79). It is also known that SICI interneurons are influenced by subcortical-to-cortical inputs (82). Thus, it is possible that rPMS-generated ascending signals participated in SICI reactivation, and this plasticity influenced in turn the control of pain and movement. The findings from a very recent basic study on Verret slices and neuromimetic simulation could provide more insights about the link between SICI increase and the clinical changes (83). Indeed, these authors demonstrated that the increase of GABA_A_ inhibition was associated with more complex brain function and wakefulness (more segregated circuits, less synchronized) as compared to no inhibition (leading to higher levels of synchronization, thus a less complex function). SICI increase in the CRPS hemisphere, after rPMS-generated massive flows of proprioceptive signals, could thus reflect a more elaborate and tuned management of the CRPS upper extremity as compared to baseline, which seems to be appropriate to better control pain and hand function.

The SICI increase in M1_contra was mirrored by a decrease of SICI in M1_ipsi, i.e., a change in the opposite direction. Tackling the functional significance of these opposite effects between the CRPS and non-CRPS hemispheres warrants further investigations. However, the presence of SICI in M1_ipsi at baseline indicates that the initial M1 disinhibition was specific to the CRPS hemisphere in our sample. Then, it is known that M1 disinhibition favors the plastic adaptation of M1 circuits (84), as shown with GABA_A_ antagonist administration (85). Thus, it is questioned whether plastic changes in the non-CRPS hemisphere (ipsilateral to the side stimulated by rPMS) could have played a role in the clinical changes. This has already been suggested in chronic stroke where rPMS applied on the paretic side reduced SICI in both hemispheres (34). The absence of brain lesion in chronic pain can lead to after-effects of rPMS different from a study in chronic stroke (24). For instance, the opposite modulation of SICI between hemispheres in CRPS but with the similar absolute hemispheric difference (Figure 3A, right part) may reflect some mechanisms of interhemispheric homeostasis. Indeed, it is known that the transcallosal fibers that wire homotopic counterparts contribute to maintain the excitation-inhibition balance needed for hemispheric function (86). One of the circuits involved here could have been an interhemispheric inhibition of SICI (82), i.e., M1_contra (activated by rPMS) reducing the SICI in M1_ipsi. Future studies should replicate these findings, test if they are transient or persistent over time, and address their functional significance in CRPS.

### Methodological Considerations

Complex regional pain syndrome is a rare disease, and the small sample size did not allow the allocation of some participants to a control group, which could have been essential to demonstrate the effectiveness of the target intervention. Therefore, the present results should be taken cautiously and should be replicated by larger sampled studies with a sham-controlled design. However, given the limited evidence to support the current treatment in CRPS (4, 5) and a challenge for larger samples in CRPS, our open-label study could provide important first insights into the therapeutic potential of rPMS in CRPS. Moreover, data reliability may have been improved by the fact that each participant was his/her own control. In addition, previous randomized double-blind placebo-controlled studies on rPMS reported that placebo did not yield any effect on the level of pain (30–32, 87). In the same vein, it should be noteworthy that the improvement of proprioception (eyes closed), the sustained pain decrease at 1 week, and the M1 changes reported could not have been due to placebo after-effects.

## Conclusion

Our open-lab pilot study provided the first ever findings on the rPMS potential to promote the CRPS condition. This study offers the advantages of concurrent clinical and brain motor outcomes from both the CRPS and non-CRPS sides tested before and after one session of rPMS of the upper limb. Brain plastic changes were obtained in parallel with significant clinical changes, especially pain reduction and improvement of upper limb proprioception and function. Importantly, it was shown that the direction of imbalanced hemispheric M1 activity matters given it influenced rPMS after-effects on pain. Future studies should be sham-control designed to replicate and confirm the existence of a causal relationship between the TMS outcomes and any rPMS-induced decrease of pain. They will have to test the after-effects of repeated sessions of rPMS over a longer period of follow-ups and to investigate, in addition to M1, the excitability changes induced in the primary somatosensory cortex. This work will contribute to the development of personalized treatments of non-invasive peripheral neurostimulation in CRPS.

## Data Availability Statement

The raw data supporting the conclusions of this article will be made available by the authors, without undue reservation.

## Ethics Statement

The studies involving human participants were reviewed and approved by the research ethics boards from the CHU de Québec and University of Laval. The patients/participants provided their written informed consent to participate in this study.

## Author Contributions

FAD conducted the study. FAD, AZ, and CS analyzed the data and wrote the paper. CS revised and edited the manuscript. All authors contributed to the article and approved the submitted version.

## Funding

This work was supported by the Canadian Foundation for Innovation (CS equipment, #10071), the Chronic Pain Network (CPN) funded by the strategy for patient-oriented research (SPOR) of the Canadian Institutes of Health Research (CS operating funds, #358108). FAD was supported by a master studentship for health professionals from the Fonds de Recherche du Québec-Santé (FRQS) and a grant research from CPN. AZ was supported by a doctorate studentship from the Centre Thématique de Recherche en Neurosciences (CTRN-Université Laval, Québec).

## Conflict of Interest

The authors declare that the research was conducted in the absence of any commercial or financial relationships that could be construed as a potential conflict of interest.

## Publisher's Note

All claims expressed in this article are solely those of the authors and do not necessarily represent those of their affiliated organizations, or those of the publisher, the editors and the reviewers. Any product that may be evaluated in this article, or claim that may be made by its manufacturer, is not guaranteed or endorsed by the publisher.

## References

[1] Borchers AT Gershwin ME. Complex regional pain syndrome: a comprehensive and critical review. Autoimmun Rev. (2014) 13:242–65. 10.1016/j.autrev.2013.10.006 24161450

[2] Marinus J Moseley GL Birklein F Baron R Maihöfner C Kingery WS. Clinical features and pathophysiology of complex regional pain syndrome. Lancet Neurol. (2011) 10:637–48. 10.1016/S1474-4422(11)70106-5 21683929 PMC5511749

[3] Goh EL Chidambaram S Ma D. Complex regional pain syndrome: a recent update. Burns Trauma. (2017) 5:2. 10.1186/s41038-016-0066-4 28127572 PMC5244710

[4] O'Connell NE Wand BM McAuley J Marston L Moseley GL. Interventions for treating pain and disability in adults with complex regional pain syndrome. Cochrane Database Syst Rev. (2013) 2013:CD009416. 10.1002/14651858.CD009416.pub2 23633371 PMC6469537

[5] Bruehl S. Complex regional pain syndrome. BMJ. (2015) 351:h2730. 10.1136/bmj.h2730 26224572

[6] Bean DJ Johnson MH Kydd RR. The outcome of complex regional pain syndrome type 1: a systematic review. J Pain. (2014) 15:677–90. 10.1016/j.jpain.2014.01.500 24530407

[7] Geertzen JH Dijkstra PU Sonderen EL Groothoff JW Duis HJ Eisma WH. Relationship between impairments, disability and handicap in reflex sympathetic dystrophy patients: a long-term follow-up study. Clin Rehabil. (1998) 12:402–12. 10.1191/026921598676761735 9796931

[8] de Mos M de Bruijn AG Huygen FJ Dieleman JP Stricker BH Sturkenboom MC. The incidence of complex regional pain syndrome: a population-based study. Pain. (2007) 129:12–20. 10.1016/j.pain.2006.09.008 17084977

[9] Rodham K Gavin J Coulson N Watts L. Co-creation of information leaflets to meet the support needs of people living with complex regional pain syndrome (CRPS) through innovative use of wiki technology. Inform Health Soc Care. (2016) 41:325–39. 10.3109/17538157.2015.1008491 25710714

[10] Barad MJ Ueno T Younger J Chatterjee N Mackey S. Complex regional pain syndrome is associated with structural abnormalities in pain-related regions of the human brain. J Pain. (2014) 15:197–203. 10.1016/j.jpain.2013.10.011 24212070 PMC4784981

[11] Geha PY Baliki MN Harden RN Bauer WR Parrish TB Apkarian AV. The brain in chronic CRPS pain: abnormal gray-white matter interactions in emotional and autonomic regions. Neuron. (2008) 60:570–81. 10.1016/j.neuron.2008.08.022 19038215 PMC2637446

[12] Kim J Loggia ML Edwards RR Wasan AD Gollub RL Napadow V. Sustained deep-tissue pain alters functional brain connectivity. Pain. (2013) 154:1343–51. 10.1016/j.pain.2013.04.016 23718988 PMC3700646

[13] Kirveskari E Vartiainen NV Gockel M Forss N. Motor cortex dysfunction in complex regional pain syndrome. Clin Neurophysiol. (2010) 121:1085–91. 10.1016/j.clinph.2010.01.032 20185362

[14] Maihofner C Handwerker HO Neundorfer B Birklein F. Patterns of cortical reorganization in complex regional pain syndrome. Neurology. (2003) 61:1707–15. 10.1212/01.WNL.0000098939.02752.8E 14694034

[15] Pleger B Ragert P Schwenkreis P Forster AF Wilimzig C Dinse H. Patterns of cortical reorganization parallel impaired tactile discrimination and pain intensity in complex regional pain syndrome. Neuroimage. (2006) 32:503–10. 10.1016/j.neuroimage.2006.03.045 16753306

[16] Schwenkreis P Janssen F Rommel O Pleger B Volker B Hosbach I. Bilateral motor cortex disinhibition in complex regional pain syndrome (CRPS) type I of the hand. Neurology. (2003) 61:515–9. 10.1212/WNL.61.4.515 12939426

[17] Vartiainen NV Kirveskari E Forss N. Central processing of tactile and nociceptive stimuli in complex regional pain syndrome. Clin Neurophysiol. (2008) 119:2380–8. 10.1016/j.clinph.2008.06.008 18723393

[18] Walton KD Dubois M Llinas RR. Abnormal thalamocortical activity in patients with Complex Regional Pain Syndrome (CRPS) type I. Pain. (2010) 150:41–51. 10.1016/j.pain.2010.02.023 20338687

[19] Zhou G Hotta J Lehtinen MK Forss N Hari R. Enlargement of choroid plexus in complex regional pain syndrome. Sci Rep. (2015) 5:14329. 10.1038/srep14329 26388497 PMC4585686

[20] Eisenberg E Chistyakov AV Yudashkin M Kaplan B Hafner H Feinsod M. Evidence for cortical hyperexcitability of the affected limb representation area in CRPS: a psychophysical and transcranial magnetic stimulation study. Pain. (2005) 113:99–105. 10.1016/j.pain.2004.09.030 15621369

[21] Krause P Forderreuther S Straube A. TMS motor cortical brain mapping in patients with complex regional pain syndrome type I. Clin Neurophysiol. (2006) 117:169–76. 10.1016/j.clinph.2005.09.012 16326140

[22] Maihofner C Baron R DeCol R Binder A Birklein F Deuschl G. The motor system shows adaptive changes in complex regional pain syndrome. Brain. (2007) 130:2671–87. 10.1093/brain/awm131 17575278

[23] Di Pietro F McAuley JH Parkitny L Lotze M Wand BM Moseley GL. Primary motor cortex function in complex regional pain syndrome: a systematic review and meta-analysis. J Pain. (2013) 14:1270–88. 10.1016/j.jpain.2013.07.004 24035350

[24] Beaulieu LD Schneider C. Repetitive peripheral magnetic stimulation to reduce pain or improve sensorimotor impairments: a literature review on parameters of application and afferents recruitment. Neurophysiol Clin. (2015) 45:223–37. 10.1016/j.neucli.2015.08.002 26363684

[25] Marinus J Perez RS van Eijs F van Gestel MA Geurts JW Huygen FJ. The role of pain coping and kinesiophobia in patients with complex regional pain syndrome type 1 of the legs. Clin J Pain. (2013) 29:563–9. 10.1097/AJP.0b013e31826f9a8a 23739533

[26] Struppler Havel P Muller-Barna P. Facilitation of skilled finger movements by repetitive peripheral magnetic stimulation (RPMS) - a new approach in central paresis. Neurorehabilitation. (2003) 18:69–82. 10.3233/NRE-2003-18108 12719622

[27] Masse-Alarie H Schneider C. [Cerebral reorganization in chronic low back pain and neurostimulation to improve motor control]. Neurophysiol Clin. (2011) 41:51–60. 10.1016/j.neucli.2011.03.004 21624706

[28] Beaulieu LD Schneider C. Effects of repetitive peripheral magnetic stimulation on normal or impaired motor control. A review. Neurophysiol Clin. (2013) 43:251–60. 10.1016/j.neucli.2013.05.003 24094911

[29] Struppler Binkofski F Angerer B Bernhardt M Spiegel S Drzezga A. A fronto-parietal network is mediating improvement of motor function related to repetitive peripheral magnetic stimulation: A PET-H2O15 study. Neuroimage. (2007) 36(Suppl. 2):T174–86. 10.1016/j.neuroimage.2007.03.033 17499165

[30] Massé-Alarie H Flamand VH Moffet H Schneider C. Peripheral neurostimulation and specific motor training of deep abdominal muscles improve posturomotor control in chronic low back pain. Clin J Pain. (2013) 29:814–23. 10.1097/AJP.0b013e318276a058 23370067

[31] Masse-Alarie H Beaulieu LD Preuss R Schneider C. Repetitive peripheral magnetic neurostimulation of multifidus muscles combined with motor training influences spine motor control and chronic low back pain. Clin Neurophysiol. (2017) 128:442–53. 10.1016/j.clinph.2016.12.020 28160750

[32] Beaulieu LD Masse-Alarie H Brouwer B Schneider C. Noninvasive neurostimulation in chronic stroke: a double-blind randomized sham-controlled testing of clinical and corticomotor effects. Top Stroke Rehabil. (2015) 22:8–17. 10.1179/1074935714Z.0000000032 25776116

[33] Beaulieu LD Flamand VH Masse-Alarie H Schneider C. Reliability and minimal detectable change of transcranial magnetic stimulation outcomes in healthy adults: a systematic review. Brain Stimul. (2017) 10:196–213. 10.1016/j.brs.2016.12.008 28031148

[34] Beaulieu LD Masse-Alarie H Camire-Bernier S Ribot-Ciscar É Schneider C. After-effects of peripheral neurostimulation on brain plasticity and ankle function in chronic stroke: the role of afferents recruited. Clin Neurophysiol. (2017) 47:275–91. 10.1016/j.neucli.2017.02.003 28314519

[35] Krewer C Hartl S Muller F Koenig E. Effects of repetitive peripheral magnetic stimulation on upper-limb spasticity and impairment in patients with spastic hemiparesis: a randomized, double-blind, sham-controlled study. Arch Phys Med Rehabil. (2014) 95:1039–47. 10.1016/j.apmr.2014.02.003 24561057

[36] Struppler Angerer B Havel P. Modulation of sensorimotor performances and cognition abilities induced by RPMS: clinical and experimental investigations. Suppl Clin Neurophysiol. (2003) 56:358–67. 10.1016/S1567-424X(09)70239-9 14677412

[37] Zhu Y Starr A. Magnetic stimulation of muscle evokes cerebral potentials. Muscle Nerve. (1991) 14:721–32. 10.1002/mus.880140806 1890997

[38] Kunesch E Knecht S Classen J Roick H Tyercha C Benecke R. Somatosensory evoked potentials (SEPs) elicited by magnetic nerve stimulation. Electroencephalogr Clin Neurophysiol. (1993) 88:459–67. 10.1016/0168-5597(93)90035-N 7694832

[39] Hasselmo ME. Neuromodulation and cortical function: modeling the physiological basis of behavior. Behav Brain Res. (1995) 67:1–27. 10.1016/0166-4328(94)00113-T 7748496

[40] Pell GS Roth Y Zangen A. Modulation of cortical excitability induced by repetitive transcranial magnetic stimulation: influence of timing and geometrical parameters and underlying mechanisms. Prog. Neurobiol. (2011) 93:59–98. 10.1016/j.pneurobio.2010.10.003 21056619

[41] Puchalski P Zyluk A. Results of the treatment of chronic, refractory CRPS with ketamine infusions: a preliminary report. Handchir Mikrochir Plast Chir. (2016) 48:143–7. 10.1055/s-0042-108650 27311072

[42] Harden RN Bruehl S Perez RS Birklein F Marinus J Maihofner C. Validation of proposed diagnostic criteria (the “Budapest Criteria”) for Complex Regional Pain Syndrome. Pain. (2010) 150:268–74. 10.1016/j.pain.2010.04.030 20493633 PMC2914601

[43] Lefaucheur JP Aleman A Baeken C Benninger DH Brunelin J Di Lazzaro V. Evidence-based guidelines on the therapeutic use of repetitive transcranial magnetic stimulation (rTMS): an update (2014-2018). Clin. Neurophysiol. (2020) 131:474–528. 10.1016/j.clinph.2019.11.002 31901449

[44] Rossi S Antal A Bestmann S Bikson M Brewer C Brockmoller J. basis of this article began with a Consensus Statement from the IFCN Workshop on "Present, Safety and recommendations for TMS use in healthy subjects and patient populations, with updates on training, ethical and regulatory issues: Expert Guidelines ≫. Clin Neurophysiol. (2021) 132:269–306. 10.1016/j.clinph.2020.10.003 33243615 PMC9094636

[45] Flamand VH Beaulieu LD Nadeau L Schneider C. Peripheral magnetic stimulation to decrease spasticity in cerebral palsy. Pediatr Neurol. (2012) 47:345–8. 10.1016/j.pediatrneurol.2012.07.005 23044016

[46] Flamand VH Schneider C. Noninvasive and painless magnetic stimulation of nerves improved brain motor function and mobility in a cerebral palsy case. Arch Phys Med Rehabil. (2014) 95:1984–90. 10.1016/j.apmr.2014.05.014 24907638

[47] Mathiowetz V Weber K Volland G Kashman N. Reliability and validity of grip and pinch strength evaluations. J Hand Surg. (1984) 9:222–6. 10.1016/S0363-5023(84)80146-X 6715829

[48] Buckinx F Croisier JL Reginster JY Dardenne N Beaudart C Slomian J. Reliability of muscle strength measures obtained with a hand-held dynamometer in an elderly population. Clin Physiol. Funct Imaging. (2017) 37:332–40. 10.1111/cpf.12300 26519103

[49] Le Métayer M L'évaluation clinique du sens de la position de l'axe du corps et des membres supérieurs par ≪l'épreuve nez et doigt≫. Motricité Cérébrale Réadaptation Neurologie du Développement. (2007) 28:25-31. 10.1016/S0245-5919(07)89941-9

[50] Rossini PM Burke D Chen R Cohen LG Daskalakis Z Di Iorio R. Non-invasive electrical and magnetic stimulation of the brain, spinal cord, roots and peripheral nerves: Basic principles and procedures for routine clinical and research application. An updated report from an I.F.C.N. Committee. Clin Neurophysiol. (2015) 126:1071–107. 10.1016/j.clinph.2015.02.001 25797650 PMC6350257

[51] Gagné M Schneider C. Dynamic changes in corticospinal control of precision grip during wrist movements. Brain Res. (2007) 1164:32–43. 10.1016/j.brainres.2007.06.014 17632089

[52] Gagné M Schneider C. Dynamic influence of wrist flexion and extension on the intracortical inhibition of the first dorsal interosseus muscle during precision grip. Brain Res. (2008) 1195:77–88. 10.1016/j.brainres.2007.12.021 18206858

[53] Hermens HJ Freriks B Disselhorst-Klug C Rau G. Development of recommendations for SEMG sensors and sensor placement procedures. J. Electromyogr. Kinesiol. (2000) 10:361–74. 10.1016/S1050-6411(00)00027-4 11018445

[54] Sakai K Ugawa Y Terao Y Hanajima R Furubayashi T Kanazawa I. Preferential activation of different I waves by transcranial magnetic stimulation with a figure-of-eight-shaped coil. Exp. Brain Res. (1997) 113:24–32. 10.1007/BF02454139 9028772

[55] Klem GH The ten-twenty electrode system of the international federation. the internanional federation of clinical nenrophysiology. Electroencephalogr. Clin. Neurophysiol. Suppl. (1999) 52:3–6. 10590970

[56] Wolf SL Butler AJ Campana GI Parris TA Struys DM Weinstein SR. Intra-subject reliability of parameters contributing to maps generated by transcranial magnetic stimulation in able-bodied adults. Clin. Neurophysiol. (2004) 115:1740–7. 10.1016/j.clinph.2004.02.027 15261852

[57] Beaulieu LD Massé-Alarie H Ribot-Ciscar E Schneider C. Reliability of lower limb transcranial magnetic stimulation outcomes in the ipsi-and contralesional hemispheres of adults with chronic stroke. Clin Neurophysiol. (2017) 128:1290–8. 10.1016/j.clinph.2017.04.021 28549277

[58] Malcolm M Triggs W Light K Shechtman O Khandekar G Rothi LG. Reliability of motor cortex transcranial magnetic stimulation in four muscle representations. Clin Neurophysiol. (2006) 117:1037–46. 10.1016/j.clinph.2006.02.005 16564206

[59] Ziemann U Reis J Schwenkreis P Rosanova M Strafella A Badawy R. TMS and drugs revisited 2014. Clin Neurophysiol. (2015) 126:1847–68. 10.1016/j.clinph.2014.08.028 25534482

[60] Devanne H Lavoie B Capaday C. Input-output properties and gain changes in the human corticospinal pathway. Exp Brain Res. (1997) 114:329–38. 10.1007/PL00005641 9166922

[61] Kobayashi M Pascual-Leone A. Transcranial magnetic stimulation in neurology. Lancet Neurol. (2003) 2:145–56. 10.1016/S1474-4422(03)00321-1 12849236

[62] Groppa S Oliviero A Eisen A Quartarone A Cohen L Mall V. A practical guide to diagnostic transcranial magnetic stimulation: report of an IFCN committee. Clin Neurophysiol. (2012) 123:858–82. 10.1016/j.clinph.2012.01.010 22349304 PMC4890546

[63] Ilić TV Meintzschel F Cleff U Ruge D Kessler KR Ziemann U. Short-interval paired-pulse inhibition and facilitation of human motor cortex: the dimension of stimulus intensity. J Physiol. (2002) 545:153–67. 10.1113/jphysiol.2002.030122 12433957 PMC2290644

[64] Arya KN Pandian S Verma R Garg R. Movement therapy induced neural reorganization and motor recovery in stroke: a review. J Bodywork Movement Therapies. (2011) 15:528–37. 10.1016/j.jbmt.2011.01.023 21943628

[65] Kujirai T Caramia M Rothwell JC Day B Thompson P Ferbert A. Corticocortical inhibition in human motor cortex. J Physiol. (1993) 471:501–19. 10.1113/jphysiol.1993.sp019912 8120818 PMC1143973

[66] Bender R Lange S. Adjusting for multiple testing-when and how? J Clin Epidemiol. (2001) 54:343–9. 10.1016/S0895-4356(00)00314-0 11297884

[67] Perneger TV What's wrong with Bonferroni adjustments. BMJ. (1998) 316:1236–8. 10.1136/bmj.316.7139.1236 9553006 PMC1112991

[68] Feise RJ Do multiple outcome measures require p-value adjustment? BMC Med Res Methodol. (2002) 2:8. 10.1186/1471-2288-2-8 12069695 PMC117123

[69] Norkin CC White DJ. Measurement of Joint Motion: A Guide to Goniometry. FA Davis (2016).

[70] Krause P Foerderreuther S Straube A. Effects of conditioning peripheral repetitive magnetic stimulation in patients with complex regional pain syndrome. Neurol Res. (2005) 27:412–7. 10.1179/016164105X17224 15949240

[71] Bilgili Cakir T Dogan SK Ercalik T Filiz MB Toraman F. The effectiveness of transcutaneous electrical nerve stimulation in the management of patients with complex regional pain syndrome: a randomized, double-blinded, placebo-controlled prospective study. J Back Musculoskelet Rehabil. (2016) 29:661–71. 10.3233/BMR-160667 26922847

[72] Bodenheim R Bennett JH. Reversal of a Sudeck's atrophy by the adjunctive use of transcutaneous electrical nerve stimulation. A case report. Phys Ther. (1983) 63:1287–8. 10.1093/ptj/63.8.1287 6192454

[73] Kesler RW Saulsbury FT Miller LT Rowlingson JC. Reflex sympathetic dystrophy in children: treatment with transcutaneous electric nerve stimulation. Pediatrics. (1988) 82:728–32. 3263617

[74] Richlin DM Carron H Rowlingson JC Sussman MD Baugher WH Goldner RD Reflex sympathetic dystrophy: successful treatment by transcutaneous nerve stimulation. J Pediatr. (1978) 93:84–6. 10.1016/S0022-3476(78)80610-6 306428

[75] Houde F Harvey MP Tremblay Labrecque PF Lamarche F Lefebvre A Leonard G. Combining transcranial direct current stimulation and transcutaneous electrical nerve stimulation to relieve persistent pain in a patient suffering from complex regional pain syndrome: a case report. J Pain Res. (2020) 13:467–73. 10.2147/JPR.S226616 32184651 PMC7060070

[76] Silva BA Astori S Burns AM Heiser H van den Heuvel L Santoni G. A thalamo-amygdalar circuit underlying the extinction of remote fear memories. Nat Neurosci. (2021) 24:964–74. 10.1038/s41593-021-00856-y 34017129

[77] Miles TS. Reorganization of the human motor cortex by sensory signals: a selective review. Clin Exp Pharmacol Physiol. (2005) 32:128–31. 10.1111/j.1440-1681.2005.04141.x 15730448

[78] Lucente G Valls-Sole J Murillo N Rothwell J Coll J Davalos A. Noninvasive brain stimulation and noninvasive peripheral stimulation for neglect syndrome following acquired brain injury. Neuromodulation Technol Neural Interface. (2020) 23:312–23. 10.1111/ner.13062 31725939

[79] Krause P Foerderreuther S Straube A. Bilateral motor cortex disinhibition in complex regional pain syndrome (CRPS) type I of the hand. Neurology. (2004) 62:1654; author reply: 1654–5. 10.1212/WNL.62.9.1654 15136713

[80] Di Lazzaro V Restuccia D Oliviero A Profice P Ferrara L Insola A. Magnetic transcranial stimulation at intensities below active motor threshold activates intracortical inhibitory circuits. Exp Brain Res. (1998) 119:265–68. 10.1007/s002210050341 9535577

[81] Byblow WD Stinear CM. Modulation of short-latency intracortical inhibition in human primary motor cortex during synchronised versus syncopated finger movements. Exp Brain Res. (2006) 168:287–93. 10.1007/s00221-005-0205-9 16328278

[82] Reis J Swayne OB Vandermeeren Y Camus M Dimyan MA Harris-Love M. Contribution of transcranial magnetic stimulation to the understanding of cortical mechanisms involved in motor control. J Physiol. (2008) 586:325–51. 10.1113/jphysiol.2007.144824 17974592 PMC2375593

[83] Barbero-Castillo Mateos-Aparicio P Dalla Porta L Camassa A Perez-Mendez L Sanchez-Vives MV. Impact of GABAA and GABAB inhibition on cortical dynamics and perturbational complexity during synchronous and desynchronized states. J Neurosci. (2021) 41:5029–44. 10.1523/JNEUROSCI.1837-20.2021 33906901 PMC8197642

[84] Keller. Intrinsic synaptic organization of the motor cortex. Cereb Cortex. (1993) 3:430–41. 10.1093/cercor/3.5.430 8260811

[85] Ziemann U Muellbacher W Hallett M Cohen LG. Modulation of practice-dependent plasticity in human motor cortex. Brain. (2001) 124:1171–81. 10.1093/brain/124.6.1171 11353733

[86] Bloom JS Hynd GW. The role of the corpus callosum in interhemispheric transfer of information: excitation or inhibition? Neuropsychol Rev. (2005) 15:59–71. 10.1007/s11065-005-6252-y 16211466

[87] Khedr EM Ahmed MA Alkady EA Mostafa MG Said HG. Therapeutic effects of peripheral magnetic stimulation on traumatic brachial plexopathy: clinical and neurophysiological study. Neurophysiol Clin. (2012) 42:111–8. 10.1016/j.neucli.2011.11.003 22500700

